# Peer perceptions of orofacial appearance among older adults – a qualitative study

**DOI:** 10.1186/s12877-026-07905-w

**Published:** 2026-06-27

**Authors:** Sara Henricsson, Viveca Wallin Bengtsson, Nina Lundegren, Pia Andersson

**Affiliations:** 1 Faculty of Health Sciences, Kristianstad University, Kristianstad, SE-291 88 Sweden; 2 Department of Oral Diagnostics, Faculty of Odontology, Malmö University, Malmö, 214 21 Sweden; 3 Department of Periodontology, Faculty of Odontology, Malmö University, Malmö, 214 21 Sweden

**Keywords:** Aging, Older adults, Oral health, Orofacial appearance, Peer assessments

## Abstract

**Background:**

Orofacial appearance remains socially meaningful throughout later life, as facial and dental features may shape first impressions and social judgments. Yet little is known about how older adults perceive the orofacial appearance of their peers. Against this background, this study explored how adults aged 65 years and older perceive their peers’ orofacial appearance (OA).

**Methods:**

Semi-structured interviews were conducted with 20 participants (aged 68–76), strategically recruited from the Swedish National Study on Aging and Care–Blekinge (SNAC-B) in Karlskrona. To identify patterns in the data, a thematic analysis was used to analyze the interviews.

**Results:**

The older adults’ perceptions of other people’s OA were represented in three themes: (i) charisma and personality overshadow orofacial appearance; (ii) it is easy to attribute characteristics as a result of the appearance; (iii) adherence to established norms is expected.

**Conclusion:**

This study highlights how older adults’ perceptions of peers’ OA are shaped by interpersonal impressions and social norms. Charisma, warmth, and facial expression could reduce the importance of specific orofacial features, whereas missing teeth, visible poor oral health, or appearance changes perceived as outside age-appropriate norms could trigger assumptions about lifestyle, vulnerability, or self-care.

**Supplementary Information:**

The online version contains supplementary material available at 10.1186/s12877-026-07905-w.

## Background

Facial and dental appearance carry significant social importance and are often the first attributes evaluated by others [1]. The appearance of teeth has been described as an important aspect of overall physical appearance and has a significant impact on a person’s self-image, social interactions, and quality of life [2, 3]. Although earlier studies have suggested that orofacial appearance (OA) may be less important in older age [4, 5], more recent body image research indicates that appearance remains relevant throughout the life course [6, 7]. Older adults may accept age-related changes while still negotiating the meaning of appearance, health, functionality, and well-being in relation to visible signs of aging [8]. This supports the relevance of focusing specifically on older adults´ perceptions of peers´ OA, as these views may be shaped by both age-related bodily changes and social expectations regarding how older adults should look [9–11]. Terms, such as facial and dental appearance, describe different features of OA. In this study, OA is defined as the individual perception of the physical characteristics of the face, mouth, and teeth, as proposed by Henricsson et al. [12].

Older adults’ perception of OA depends on factors such as self-perception [12], internalized age stereotypes [13, 14], societal constructs [15] including societal standards of beauty, and personal experiences [16]. According to the “stereotype embodiment theory”, internalizing cultural and societal stereotypes contributes to the formation of self-perception of aging, impacting both health and functionality [13, 14], which can influence how older adults view attractiveness in their peers [16]. Societal attitudes also play an important role in shaping perceptions of appearance and of aging [11]. The stereotypical portrayal of older adults may influence how they perceive themselves and their peers [15]. Societal emphasis on youthfulness often shapes older adults’ perceptions of attractiveness [11]. The societal construct, where aging has a more negative impact on perceptions of women’s attractiveness compared to men’s, can also influence how older adults view each other, with older women potentially being judged more harshly based on appearance than older men [17].

A recent interview study showed that older adults hold dual perceptions of their own OA: although a younger appearance was considered socially beneficial, the active pursuit of a youthful appearance was viewed negatively [12]. In that study, nice teeth were described as an important aspect of OA, but informants’ own teeth were often overlooked in favor of other facial attributes unless oral health or teeth were perceived as problematic [12]. These findings illustrate the nuanced and sometimes contradictory ways in which older adults evaluate their own OA. Older adults’ perceptions of OA are shaped by societal norms [9, 11] and their own self-image [12]. However, self-perception and peer perception are not necessarily shaped in the same way. Evaluations of others OA may involve social and normative dimensions that are of minor importance when individuals reflect on their own appearance.

Although previous research has examined older adults’ perceptions of their own OA, little is known about how they evaluate their peers’ OA or how such evaluations are shaped by social norms and age-related expectations. Moreover, findings from younger populations or studies of general facial attractiveness cannot be directly transferable to older adults, whose perceptions may be shaped by life experience, age stereotypes, expectations of “normal” aging, and changing oral health conditions. Exploring peer perceptions of OA may therefore contribute new knowledge about how appearance-related norms operate in later life and may help dental professionals understand social meanings attached to dental status, dentures, and visible oral health among older adults. In this study, the aim was to explore how adults aged 65 years and older perceive their peers’ orofacial appearance.

## Methods

### Study design

This study employed an exploratory qualitative interview design with strategic sampling. The design was chosen because the aim was to gain an in-depth understanding of older adults’ perceptions and interpretations of their peers’ OA, a topic that is relatively underexplored. Individual semi-structured interviews were used to allow informants to describe sensitive or potentially socially undesirable views, such as prejudice or discomfort in relation to others’ appearance, in a private setting. Focus groups were therefore considered less appropriate because group interaction could have reinforced socially acceptable responses or inhibited discussion of stigma and judgement. Thematic analysis with elements of reflexivity, following Braun and Clarke [18], was used to identify and interpret patterned meanings across the interview data. This approach was appropriate because the study aimed not only to summarize what informants noticed in peers’ OA but also to interpret how these perceptions were shaped by social norms, age-related expectations, and moral self-reflection. The study adheres to the COREQ (Consolidated Criteria for Reporting Qualitative Research) checklist [19] (available as a supplementary file).

### Recruitment and participants

The participants were selected from the SNAC-B study in Karlskrona, Sweden, which is one of four research centers in the ongoing Swedish National Study on Aging and Care (SNAC). SNAC was established in 2001 to better understand aging, focusing on the transition to retirement and later life [20]. Invited participants undergo re-examinations every six years from age 66 until they reach 78 years old, and every three years thereafter. The SNAC-B study is described in more detail elsewhere [12, 21, 22].

Twenty strategically recruited participants from the SNAC-B registry follow-up 2019–2021 were included in this study. A strategic sampling procedure was applied to achieve variation in age, sex, and number of teeth, as these characteristics were considered relevant to experiences and perceptions of OA. Number of teeth was used as a sampling criterion because dental status could influence both how informants perceived their own OA and how they reacted to the dental appearance of others. Besides being proficient in Swedish, participants should be between 65 and 79 years of age to participate in the study. Based on these criteria, the five men and five women with the highest and lowest numbers of teeth were selected from a coded list of participants aged 66 and 72 years in the 2019–2021 SNAC-B follow-up. At the time for the interview, the informants were 68 to 76 years old, with an average age of 72 years. The included informants were the same as in a previous study on self-perceived OA [12], but two separate interviews were conducted, each guided by the distinct aims of the respective studies. See Table 1 for descriptions of the participants, who will henceforth be referred to as informants.

**Table 1:** Description of included informants

ID:	Gender	Age	Place of birth	Living arrangement	Educational level	Cash margin The ability to get 15,000 SEK within one week	Presence of perceived/ previously perceived dental issues	Perceived general health
I:1	Male	68	Sweden	Living with someone	˃ 9 years of schooling	Yes	None	Good/excellent
I:2	Female	68	Other European country	Living alone	˃ 9 years of schooling	Yes	Significant	Poor/fair
I:3	Female	74	Sweden	Living with someone	˃ 9 years of schooling	-	Minor	Good/excellent
I:4	Female	69	Other European country	Living alone	˃ 9 years of schooling	Yes	Minor	Good/excellent
I:5	Female	68	Other European country	Living with someone	˃ 9 years of schooling	Yes	Significant	Good/excellent
I:6	Male	68	Sweden	Living with someone	˃ 9 years of schooling	-	None	-
I:7	Male	76	Sweden	Living with someone	˃ 9 years of schooling	Yes	Significant	Good/excellent
I:8	Male	74	Sweden	Living with someone	˃ 9 years of schooling	Yes	Minor	-
I:9	Female	76	Sweden	Living alone	˃ 9 years of schooling	Yes	Significant	Good/excellent
I:10	Female	69	Sweden	Living with someone	-	Yes	Minor	Good/excellent
I:11	Male	74	Sweden	Living with someone	˃ 9 years of schooling	-	None	-
I:12	Female	76	Sweden	Living with someone	≤ 9 years of schooling	No	None	Good/excellent
I:13	Female	76	Sweden	Living with someone	˃ 9 years of schooling	Yes	Minor	Good/excellent
I:14	Male	76	Sweden	Living with someone	˃ 9 years of schooling	Yes	Minor	Good/excellent
I:15	Male	68	Sweden	Living alone	˃ 9 years of schooling	Yes	None	Good/excellent
I:16	Male	68	Sweden	Living with someone	˃ 9 years of schooling	Yes	None	Poor/fair
I:17	Female	69	Sweden	Living with someone	˃ 9 years of schooling	No	None	Good/excellent
I:18	Male	74	Sweden	Living with someone	≤ 9 years of schooling	Yes	Minor	Good/excellent
I:19	Male	69	Sweden	Living with someone	˃ 9 years of schooling	Yes	Significant	Good/excellent
I:20	Female	74	Sweden	Living with someone	˃ 9 years of schooling	Yes	Significant	Poor/fair

The informants received detailed information about the study by mail, followed by a phone call from the first author (SH) within a week, with a request to participate. Of the 20 initially invited, four declined, and one was unable to attend. In line with the previous procedure, mail was sent to five new persons, who all agreed to participate.

### Data collection and procedure

During May and June 2023, individual interviews were conducted at the research clinic of Blekinge Institute of Technology. Before the interview, informants were asked to consent to the use of electronic recording devices.

A semi-structured interview guide was developed by the authors for the interviews (available as a supplementary file). Following the pilot interviews, minor modifications were made to the interview guide for clarification purposes. The questions in the interview guide addressed informants’ opinions about their peers’ OA. Probing questions were used to clarify, enrich, or deepen participants’ answers when necessary. Examples of probing questions were, “Could you give an example?” or “Could you explain that further?”.

**Fig. 1:**
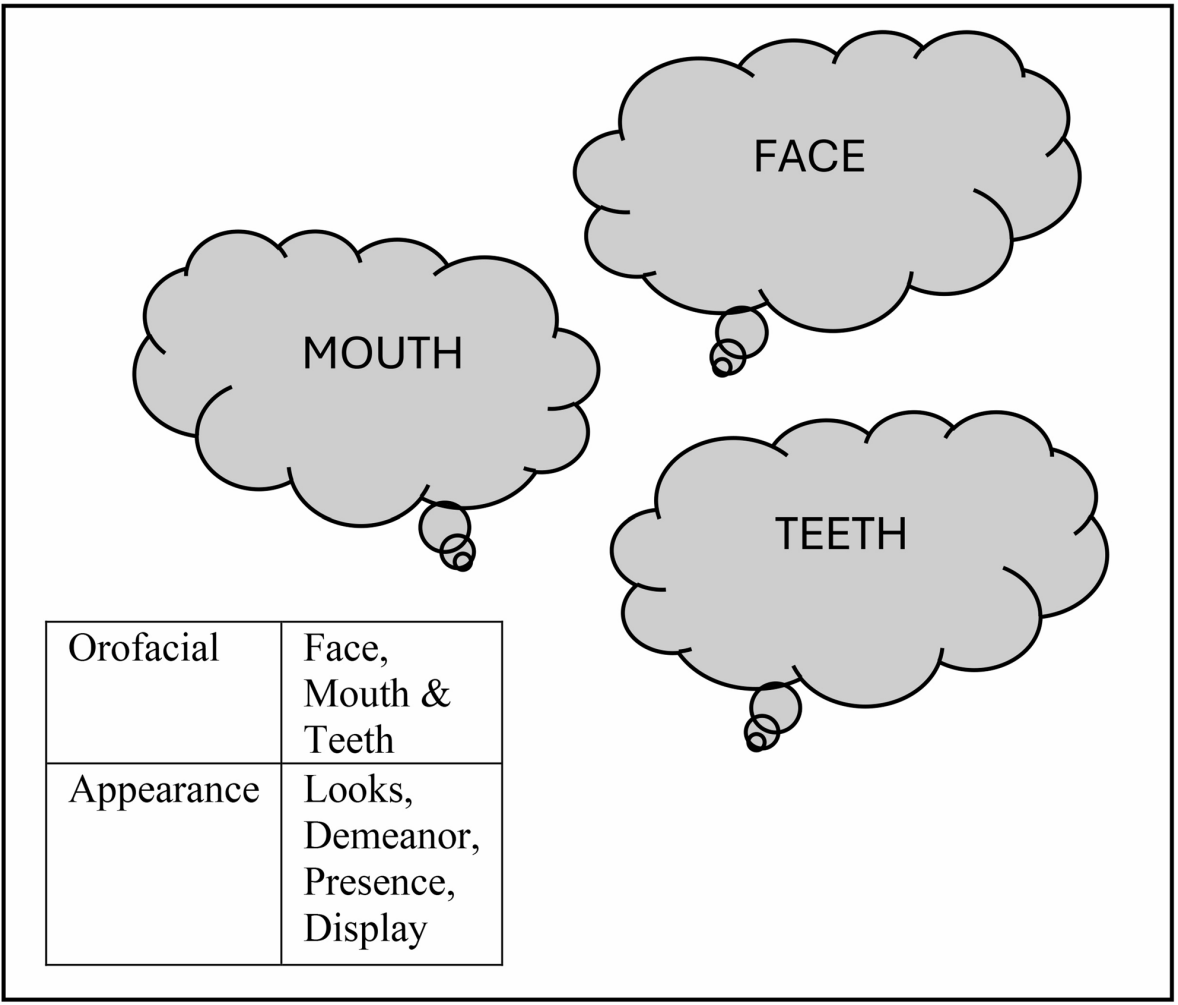
Perception of physical characteristics of the face, mouth, and teeth

Prior to the interview, informants were informed that the primary aim of the interview was to elicit their perceptions of OA in their peers. During the interview, a document containing the definition of OA and synonyms for the word appearance (Fig. 1) was placed in front of them to facilitate reflection on the subject in focus. All interviews were conducted by the first author (SH), with support from the last author (PA). Neither of them was known to the informants. The interviews were transcribed verbatim and had an average duration of 24 min (ranging from 16 to 38 min).

### Data analysis

The interview data were analyzed using thematic analysis employed by Braun and Clarke [18] to identify, analyze, and interpret patterns of meaning within qualitative data. Following Braun and Clark [18], the analysis was conducted in six phases. In the first phase (1) *familiarization with the data and writing familiarization notes*, all authors (SH, PA, NL, VWB) read and re-read the transcripts, made notes on emerging patterns, and discussed their initial impressions. In the second phase (2), *systematic data coding*, the first author (SH) conducted the initial coding in NVivo Release 1.7.1 (QSR International Pty Ltd.), which was used as a data-management tool to store transcripts, organize codes, retrieve coded extracts, and facilitate comparisons across interviews. Code lists, coded extracts, and preliminary theme structures were then exported and shared with all authors for repeated discussion. During the third phase (3) of *generating initial themes from coded and collated data*, the first author identified potential themes by combining codes with shared characteristics and discussing them with all authors. Preceding the fourth phase (4) of *developing and reviewing themes*, authors individually revisited codes and initial themes to develop and review the themes. In the fifth phase (5), the themes were *refined*,* defined*,* and named* through collaborative discussion. In the final phase, *writing of the report* (6), SH synthesized the findings into a coherent narrative and selected representative quotations for each theme [18]. All authors contributed to the interpretation of the data and to the refinement of themes, although only SH conducted the coding in NVivo. All authors had previous experience in conducting and publishing qualitative research. Any disagreements or alternative interpretations were discussed in author meetings until the themes were considered coherent and grounded in the data. Examples of the identified codes and themes are presented in Table 2.

**Table 2:** Examples of codes and themes

Citation	Code	Theme
*“And then you meet someone whose face is not*,* like*,* picture-perfect*,* but you really like them*,* and then suddenly… if you were to describe them*,* you would do it in positive terms*,* like… Oh yeah*,* her!” (I:10)*	Reassessment of a first impression	Charisma and personality overshadow orofacial appearance
*“Yeah*,* if someone comes up to you without their front teeth*,* and they’re not a hockey player*,* you wonder how they lost them. I mean*,* you just… you naturally think about that*,* you know?” (I:14)*	Creates an image of a person based on their OA	It is easy to attribute characteristics as a result of the appearance
*“Well*,* you know*,* it is a person who… who looks fresh*,* you know. Not all painted up to death and all that. Cause that does not look fresh to me. Just*,* you know*,* wears normal makeup*,* if it is a woman*,* or if it is a man*,* … is reasonably clean-shaven*,* maybe a little stubble*,* that’s alright .//. But that… that is what it is really about*,* right? That they do not make a big show of themselves.” (I:18)*	Other factors that influence how OA is perceived	Adherence to established norms is expected

## Results

The thematic analysis of older adults’ perceptions of their peers’ OA generated three themes:

- *Charisma and personality overshadow orofacial appearance; it is easy to attribute characteristics as a result of the appearance; and adherence to established norms is expected.*

Across the three themes, informants’ perceptions of their peers’ OA were characterized by a recurring tension between an expressed ideal of accepting others regardless of appearance and spontaneous reactions shaped by socially learned norms. The three themes are therefore interrelated rather than separate categories: charisma could soften or override attention to OA, visible deviations could trigger assumptions about character or lifestyle, and norms of age-appropriate appearance set boundaries for what was considered acceptable.

### Theme 1 - charisma and personality overshadow orofacial appearance

Charisma and perceived warmth can override visible orofacial features, suggesting that OA is interpreted through the person’s social presence rather than through appearance alone.

The first impression of another person was, in different ways, shaped by the overall OA, but this impression was quickly filtered through the person’s social presence. Informants described how warmth, eye contact, and a friendly expression could make orofacial details less noticeable, whereas a tense, neutral, or unfriendly expression could make the face feel distant and harder to connect with. In this sense, OA was not evaluated as a set of isolated features, but as part of the person’s perceived social openness.

*“So, it’s like.. you take in the whole face ..//.. then you eventually start noticing other details” (I:1)*

The informants highlighted the importance of harmony between facial features for the overall impression of OA. Specific features such as the teeth could attract attention, especially when they disrupted the sense of harmony in the face or when they were difficult for the informant to ignore. Teeth therefore mattered, but mainly when they became visually prominent or when the informants perceived them as unkempt, discolored, or missing.

*“…if someone has got an underbite*,* I just can not help but look. It is just… it bugs me somehow. Poor person*,* for me to think that*,* but it just bugs me. …//… Yeah*,* it is just the fact it sticks out. I do not know why*,* but that’s how it is.” (I:20)*

At the same time, informants emphasized that what ultimately made another person appealing was not the appearance itself, but the person’s charisma. They associated an appealing OA with personality traits such as being cheerful, positive, friendly, or kind. This also applied to specific features, such as kind eyes or a cheery smile. A pleasant and welcoming demeanor could soften the impact of visible imperfections and make the person appear more attractive overall. Conversely, a neutral, disinterest, or unfriendly facial expression could diminish the impression of OA, even when no obvious physical flaw was present.

*“Obviously a good look is great… I mean*,* it is positive in the sense that when you meet someone and I feel like … I feel like they are happy and have a way about them*,* a smile*,* eye contact and all that*,* meeting them just hits you in a good way. And then I do not think I am all that hung up on what they look like—I think it is more about their energy*,* you know” (I:6)*

*“And then you meet someone whose face is not*,* like*,* picture-perfect*,* but you really like them*,* and then suddenly… if you were to describe them*,* you would do it in positive terms*,* like… Oh yeah*,* her!” (I:10)*

In this respect, OA appeared to be seen less as a fixed physical trait but more as part of the interpersonal impression, where warmth and social ease could outweigh visible orofacial features. For some informants, the mouth and teeth were especially important, whereas the eyes and facial expression carried greater weight for others. A smile or direct eye contact could create a feeling of connection and make the person appear friendly. Thus, what was noticed first was not uniform across informants, but the final impression was shaped by the degree to which the person was perceived as socially open and engaging.

*“…I see their facial expressions*,* like*,* and… yeah*,* their whole vibe toward me as a person. That is what I notice.” (I:1)*

### Theme 2 - it is easy to attribute characteristics as a result of the appearance

Although the informants emphasized that people should not be judged by their appearance, they often described immediate appearance-based impressions, showing a tension between moral intent and spontaneous social evaluation.

Informants described how they often formed an immediate image of another person based on OA, and how this image could extend beyond appearance to assumptions about personality, social behavior, or self-care, for example *“if there is only one tooth left in the mouth… you know that there has been a lot of alcohol going on there” (I:18).* These judgments were often expressed spontaneous. Informants acknowledged that beauty standards and perceptions of aging could influence how they interpreted other people’s appearance. Several of them recognized that appearance-based judgments could occur even when they knew such judgments were unfair.

*“It is like*,* you know*,* you notice different things… stuff you think looks nice and stuff that doesn’t… you build up an image of a person. I guess you do it unconsciously. …//… And I think it is because of TV and the media*,* you know” (I:20)*

Although many informants emphasized that people should not be judged by appearance alone, they also described how difficult it was to avoid such first impressions. Some reacted internally but tried not to show it, while others admitted that a poor dental appearance could cause them to behave more cautiously or reservedly. In this way, a tension was revealed between an explicitly stated norm of fairness and an implicit tendency to make immediate appearance-based inferences. However, after further interaction with this person, that first image or reaction could be re-evaluated afterwards.

*“…if you come across someone who is missing their two front teeth*,* I do notice it*,* but I do not react—or at least*,* I absolutely do not show it. I never let it show.” (I:11)*

Informants described the tension between a normative commitment not to judge others by appearance and the immediate, spontaneous first impressions that were created. These “snap impressions” were often experienced as difficult to control and were recognized as potentially unfair, yet they still shaped how peers were perceived initially. In this sense, OA was not only a visual feature but also provided a cue that triggered moral self-awareness about their own judgments.

*“Yeah*,* it is not exactly flattering that we judge the way we do. Or that we have those snap impressions—whether positive or negative—it is kind of scary… But at the same time*,* it is just that spontaneous reaction you feel.” (I:8)*

These reactions were particularly strong when OA suggested poor dental health or visible neglect. Missing teeth, damaged teeth, or an unkempt appearance raised questions but also some skepticism about what the underlying cause might be, with some causes considered ‘better’ than others. Some informants described greater sympathy when the change seemed accidental or medically related, whereas self-inflicted or lifestyle-related consequences stemming from poor lifestyle choices were viewed more critically. Poor dental appearance could therefore function as a cue for broader social categorization.

*“If someone has a disability in the face of some kind*,* then you probably get a bit more guarded and wonder*,* oh…what has happened?” (I:4)*

*“…then I probably just look at their teeth when they speak and wonder why they have not had new ones put in or whatever. I guess I would not trust someone like that as much. Unfortunately.” (I:17)*

Some informants also linked visible dental problems with vulnerability, lower socioeconomic status or a fear of being seen in the same way themselves. Their reactions were not only directed toward others; they also reflected an awareness of how easily they themselves could be judged through appearance. This made OA a socially charged feature, carrying meaning beyond physical form.

*“…I feel like—I definitely do not want to be seen like him or her. It is just that you do not want to be like that yourself. It is just… yeah*,* they might look at me the same way I think of them or look at them. Like… well*,* here he comes…. and he is missing a couple of teeth in the front*,* or—yeah. Or has some broken teeth. It…… no*,* it is something that I……. I do not want to look like that in other people’s eyes.” (I:11)*

### Theme 3 - adherence to established norms is expected

Informants’ evaluations of OA were shaped by a normative standard of what appeared natural, well-groomed, and age-appropriate, while deviations from this standard were more likely to trigger skepticism.

Informants described “normal” OA as an appearance that was well maintained, socially familiar, and consistent with expectations of aging. This did not mean looking young or perfect; rather, it meant avoiding features perceived as neglected, artificial, exaggerated, or outside established gendered and age-related norms. Negative reactions were triggered by poor oral health, but also by how visible changes were interpreted: as understandable aging or misfortune, as lack of self-care, or as deliberate nonconformity. In this sense, normality was negotiated through the meaning assigned to visible changes rather than through appearance alone.

*“…you just have to expect that wrinkled appearance at that age*,* basically. It is not something that really displeases me*,* you know? You do your best to look as appealing as possible… given your age and all. Mm-hmm.” (I:8)*

Some informants described a clear boundary between acceptable aging and attempts to modify appearance in ways they regarded as excessive. Cosmetic alterations that were perceived as overdone could evoke skepticism or even be perceived as ridicule, especially when they were seen as overt attempts to look younger rather than as modest maintenance of appearance. What was considered appropriate was therefore not simply a matter of attractiveness, but of staying within the limits of what was perceived as natural and age-consistent.

*“…when someone has had work done on their face*,* I find it hard not to look at those pumped-up lips or when their face is so frozen so they cannot move their face or something like that. You notice those artificial oddities…//… Those who try to change their appearance—uh—to look younger often end up looking ridiculous or blatantly odd…. They think they are making themselves more attractive. But in many cases*,* they just make a fool of themselves instead.” (I:16)*

This same normative standard also applied to dental appearance. Missing teeth, dentures, or visibly neglected oral care could trigger unease or skepticism, especially when they were interpreted as signs that the person had not taken proper care of themselves. At the same time, sudden or unexpected deviations from what was considered normal could be especially salient, including when a person known to wear dentures appeared without them.

*“I saw my brother the other day and he had… he wears dentures up front*,* and he had taken them out*,* as you can do. And I got really scared. It looked really nasty. So yeah*,* something like that can definitely affect you…” (I:7)*

The informants also described that appearance should remain within a socially acceptable frame, and that certain forms of self-presentation could be interpreted as excessive or inappropriate. This was not only about facial or dental features but also about broader judgments regarding what older adults, men, and women should look like. In this way, OA became linked to expectations about social norms concerning age and gender, as well as to the boundaries of acceptable self-presentation.

## Discussion

The main finding of this study was that older adults’ perceptions of peers’ OA were shaped by a tension between moral ideals of non-judgement and socially learned norms about appearance, aging, and self-care. Informants often emphasized that personality, warmth and charisma were more important than physical appearance, and they stated that people should not be judged on the basis of OA. At the same time, they described spontaneous reactions to missing teeth, poor oral hygiene, cosmetic alterations, or other features perceived as outside the boundaries of “normal” and age-appropriate appearance. The third theme does not represent the same duality as the first two themes; rather, it shows the normative frame within which the duality becomes visible. Together, the three themes illustrate how peer perceptions of OA in later life are negotiated between acceptance, stigma, age norms, and moral reflexivity.

The findings also clarify what “normality” meant for the informants. Normality was not described as dental or facial perfection, but as an appearance that fitted expectations of the current age, self-care, and social context. Wrinkles or age-related changes were largely accepted when interpreted as part of normal aging, whereas missing teeth, visibly poor oral hygiene, exaggerated cosmetic procedures, or facial modifications could be interpreted as signs of neglect, vanity, deviance, or poor judgment. Importantly, negative responses were strongest when the change was interpreted as self-inflicted or preventable. This suggests that stigma was not attached to oral or facial difference uniformly; it depended on whether the difference was understood as misfortune, illness, aging, lack of self-care, or deliberate nonconformity. The present findings extend previous research on older adults’ self-perceived OA [12] by showing that age-appropriateness and naturalness were not only used when informants reflected on their own appearance but also when evaluating peers. In this sense, “normality” appeared to be negotiated in relation to age, perceived naturalness, and whether changes were interpreted as acceptable or excessive [8–11]. This suggests that peer assessments of OA were not based solely on visible orofacial features, but also on social norms regarding appropriate appearance in later life.

The informants highlighted the overall harmony and charisma of the face rather than focusing on individual orofacial features. Existing research on how older adults perceive their peers’ OA has not focused on specific facial features. Instead, the emphasis lies on how aging impacts perceptions of facial attractiveness [23, 24], how social factors influence age-related facial preferences [25] and how facial expressions are linked to personality traits [26, 27]. Altogether, this suggests that the overall attractiveness of the face is not determined by a single feature, but rather by the interaction of all facial features, which is also consistent with Tatarunaite et al. [28], although they have studied a younger population.

Informants noticed when features disrupted facial symmetry, such as an underbite or a crooked mouth. Such traits can evoke stereotypes—for example, under-bites are sometimes associated with aggression [29]. Still, even when one feature was more prominent than another, informants emphasized positive aspects of their peers’ OA, provided their appearance was within the bounds of what was considered ‘normal’ and age-appropriate, and not self-inflicted. This may reflect the phenomenon known as the ‘positivity effect’ [30], whereby older adults tend to emphasize positive attributes, perceiving their peers’ OA more favorably by highlighting positive expressions and downplaying deficiencies. This was apparent in that informants tended to disregard the orofacial deviations in peers they perceived to have a positive charisma. However, for those deemed to be outside the range of ‘normal’, strict norms of appearance were still maintained. It has been shown that older adults have a stronger halo effect for faces that are closer to their age [31]. The ‘halo effect’ refers to positive attributes in one area influencing perceptions in another [32]. A person’s charisma can thereby positively influence perceptions of their overall harmony of the orofacial features.

Informants emphasized that warmth and friendliness in personality enhanced their perception of OA, often making facial and oral imperfections less noticeable. A pleasant and welcoming demeanor was perceived to enhance the features of OA, making the person more appealing and attractive than it would have done otherwise. In cultures across the world, socially desirable personality traits are positively associated with attractiveness, while socially undesirable traits are linked to lower attractiveness [32]. Accordingly, Kotter-Grühn and Hess [33] found that higher attractiveness ratings correlated with perceptions of other positive traits (physical and cognitively fit, likeability, etc.). This link between attractiveness and positive qualities (likeability, competence, etc.) was demonstrated by Dion et al. [34] over 40 years ago.

Informants’ perceptions of peers’ OA were often shaped by internalized beliefs, social norms, and media portrayals. Loos et al. [15] highlight the substantial role of media representation and societal norms in shaping perceptions of aging and appearance. Older adults are often confronted with stereotypical representations, impacting how they perceive themselves and others [15]. Informants were aware that they were likely to have prejudices, probably influenced by today’s media-driven world. However, judgments of personality traits such as sociability are made based on the appearance of the face because emotional expressions are considered to be reflected in the face [35]. According to Jaeger et al. [36] perceptions of the personality traits reflected in facial expressions are related to the extent to which people rely on facial impressions. As mentioned earlier, individuals often associate attractive features with positive personality traits [35, 37], and vice versa [35]. Poor dental appearance could lead others to judge people negatively on a range of personality traits. It was not uncommon for informants in the present study to see a feature in the OA of others that they themselves struggled with, causing them to judge their peers negatively. According to Newton et al. [38], participants dissatisfied regarding tooth color exhibited more stereotypical behaviors than satisfied participants by judging dark and natural teeth lower in favor of whitened teeth [38]. It is plausible that dissatisfaction with other features in OA also exhibits stereotypic behaviors, although there is no support for this in the literature. The discrepancy between informants’ expressed view that people should not be judged by appearance and their own reported reactions may be understood as moral reflexivity.

The findings suggest that peer evaluations of OA were not only aesthetic assessments but also involved moral interpretations. This was particularly evident when visible dental problems were interpreted in relation to lifestyle, hygiene, or personal responsibility. Dental appearance affects judgments of facial attractiveness [39]. The informants reacted with a variety of emotions and behaviors when their peers had bad or missing teeth — from concern and empathy to social discomfort. While some informants saw it as a normal part of aging, others felt uneasy or uncomfortable around those with poor oral health. Not being able to afford dental care was not considered to be related to low socioeconomic status by the informants in this study, as mentioned by Willis et al. [40]. Instead, socioeconomic status was related to factors such as poor (oral) hygiene or the individual’s lifestyle choices. Facial difference (FD) can be defined as “a face whose characteristics make it deviate significantly from what an individual who perceives it expects from a normal human face” [37] and is often stigmatized. This concept is especially relevant when interpreting how missing teeth or asymmetries affect perceived status. Stigma can also affect people who do not have FD themselves but are somehow associated with someone who has FD through “stigma by association” [37]. Some informants did associate missing or bad teeth with lower socioeconomic status, triggering strong emotional reactions and fear of being judged similarly. Individuals with one or more missing upper front teeth have been perceived more negatively across various social traits than persons with full dentition, particularly when participants imagined close relationships with the individual, such as dating them or living as neighbors [40]. Research directly addressing how older adults view peers with poor oral health in relation to socioeconomic status is limited, with most studies focusing on self-perception instead of peer assessment. However, some studies provide insight into the social implications of oral health among older adults, which may indirectly contribute to our understanding of peer perceptions [41, 42], but also that research applied to younger people can be transferred to older adults.

Few studies have explored how older adults associate OA with gender roles or cosmetic modifications perceived as unnatural. However, evidence suggests it is more socially acceptable to look older than to appear “unnatural” [10].

### Clinical relevance

The findings suggest that visible dental conditions, such as missing teeth, dentures, and visibly poor oral health, may carry social meanings beyond oral function, including assumptions about lifestyle, socioeconomic vulnerability, and self-care. In encounters with older adults, dental professionals should therefore be aware that appearance-related concerns reflect not only esthetic preferences but also aspects of normality, vulnerability, and how one is perceived by others. A person-centered approach that acknowledges both function and appearance may help clinicians respond more sensitively to older patients’ concerns about oral health and OA.

### Strengths and limitations

To ensure the interview sample yielded sufficient information, Braun and Clarke [18] suggest using the information power model developed by Malterud et al. [43]. Based on the aforementioned information power model [43] 20 informants were deemed adequate considering the broad research aim, less targeted participant selection, and a previously undefined topic. In retrospect, the sample size was justified, as the topic was unfamiliar and sometimes difficult to discuss. It was also relevant regarding specificity because, despite strategic recruitment emphasizing specific variations, the sample was not directly relevant to the study’s aim [43]. In order not to influence the outcome of the interview, the informants were informed about the subject of the study, but not about the fact that the interviewer was a registered dental hygienist. If the informants had known this, they might have focused more on teeth than they did. However, the chosen approach ought to have strengthened the result’s credibility.

The transferability of the findings should be considered in relation to the sample characteristics and study context. Most informants were born in Sweden, and the study was conducted in a Swedish medium-sized municipality. Cultural background, socioeconomic position, and educational level may influence perceptions of OA, including norms related to tooth alignment, tooth color, aging, and acceptable appearance [11, 44, 45]. Although the sample was strategically selected for variation in age, gender, and number of teeth, it was not designed to capture cultural or socioeconomic diversity. This limits the possibility of interpreting how classed, cultural, or ethnic norms shaped perceptions of OA, and should be considered when assessing the transferability of the findings to other cultural, social, or clinical contexts [43]. In addition, the same informants participated in a previous study on self-perceived OA [12] in which they had already discussed their own OA. This may have helped them reflect on the topic, but it may also have shaped how they spoke about peers.

Throughout the analysis, the authors discussed their assumptions and interpretations. By considering the criteria of credibility, transferability, dependability, and confirmability, a study’s trustworthiness can be determined [46]. Credibility was strengthened through interviewer debriefing after each interview, repeated readings of the transcripts, team discussions of preliminary codes and themes, and the use of illustrative quotations that connect interpretations to the empirical material. Transferability was supported by describing the study context, recruitment process, participants, and analytic procedures so that readers can assess relevance to other settings. Dependability was enhanced by using the same semi-structured interview guide, collecting data within a limited timeframe, employing Braun and Clarke’s [18] step-by-step thematic analysis, and documenting the analytic steps from coding to theme development. Confirmability was supported through author discussions in which alternative interpretations of codes and themes were considered, including the researchers’ reflections on the analysis. To achieve confirmability, data extracts of participants’ responses were provided, with each quotation referencing its originating interview.

## Conclusion

This study highlights how older adults’ perceptions of peers’ OA are shaped by interpersonal impressions and social norms. Charisma, warmth, and facial expression could reduce the importance of specific orofacial features, whereas missing teeth, visible poor oral health, or appearance changes perceived as outside age-appropriate norms could trigger assumptions about lifestyle, vulnerability, or self-care.

## Supplementary Information

Supplementary Material 1.

Supplementary Material 2.

## Data Availability

The data presented in this study is available upon reasonable request from the corresponding author. The data is not publicly available due to privacy or ethical restrictions.

## Abbreviations

COREQ: Consolidated criteria for reporting qualitative research
OA: Orofacial appearance
SNAC-B: Swedish National Study on Aging and Care, Blekinge
