# Supplementary material for: Peer perceptions of orofacial appearance among older adults – a qualitative study
Source: BMC Geriatr. 2026 Jun 27;26:877. doi: 10.1186/s12877-026-07905-w (PMC13312759; doi:10.1186/s12877-026-07905-w)
Supplement: Supplementary file 1 — Supplementary Material 1. [file 12877_2026_7905_MOESM1_ESM.docx]

| Interview guide |  |
| --- | --- |
| How do you perceive the OA of other people your own age? |  |
| When you meet someone for the first time, what aspects of their OA do you notice? |  |
| When a person makes a positive impression on you, what aspects of their OA contribute to that impression? |  |
| What aspects of a person’s OA may contribute to a negative impression? |  |
| In what ways, if any, can a person’s OA influence how you interact with or respond to them? |  |
| How might a more or less appealing OA influence a person’s opportunities or social advantages? |  |
| How do minor esthetic imperfections of the teeth or visible tooth loss influence your perceptions or reactions, if at all? |  |
| When you meet someone with a visibly damaged or missing tooth, what thoughts or reflections come to mind? |  |
| Examples of probing questions:  - Could you give me an example?  - Could you explain that further?  - In what way has it affected you? |  |
